# Dysregulation of over-expressed IL-32 in colorectal cancer induces metastasis

**DOI:** 10.1186/s12957-015-0552-3

**Published:** 2015-04-12

**Authors:** Yi Yang, Zihao Wang, Yiming Zhou, Xiaoxiao Wang, Jianbin Xiang, Zongyou Chen

**Affiliations:** Department of General Surgery, Huashan Hospital Affiliated to Fudan University, Floor 16, Building 2, 12 Wulumuqizhong Road, Shanghai, 200040 China

**Keywords:** IL-32, Colorectal cancer, Metastasis

## Abstract

**Background:**

Interleukin (IL)-32 is a described intracellular pluripotent pro-inflammatory mediator, characterized by the signaling of NF-κB and STAT3.

**Methods:**

Our study investigated whether IL-32 expression has clinical significance in the metastases of colorectal cancer (CRC). A total of 70 CRC patients were enrolled, 47 cases of which were single CRC organic metastasis lesions while the rest of which were primary CRC lesions (T4NxM0). IL-32 expression was detected by immunohistochemistry, and the correlation between IL-32 expression and CRC metastases was analyzed.

**Results:**

The positive rates of IL-32 in the CRC organic metastasis group were more severe than those in the primary CRC group (*P* < 0.05). The positive rate of IL-32 in primary CRC with lymph node metastasis was more severe than that of IL-32 in primary CRC without lymph node metastasis (*P* < 0.05).

**Conclusions:**

The level of IL-32 expression could influence the *N* grade of CRC. Thus, IL-32 expression may stimulate the organic metastasis and the lymph node metastasis of CRC.

## Background

Colorectal cancer (CRC) is the one of the most common malignancy tumor worldwide and the fourth common cause of death of cancer [1]. It is estimated that CRC will cause more than 500,000 all over the world in 2013 [2]. A large amount of patients are initially diagnosed with develop stage IV CRC [3]. With the therapeutic development for CRC, the overall survival keeps on increasing, but the treatment of patients with metastasized CRC still remains a challenge [4,5], and the underlying mechanism is actually unsolved [6,7]. As a result, it is always the main focus of clinical medicine to find the biomarker of CRC metastasis.

Cytokines are known to be involved in not only inflammation but also tumor development and progression [8]. Several researches have suggested that many cytokines (such as IL-10, IL-17, IL-22, IL-23, and IL-35) may have clinical significance in CRC [9-11].Interleukin-32 (IL-32) is recognized as an intracellular pluripotent pro-inflammatory mediator firstly [12]. IL-32 can stimulate immunosuppressive immunocytes and lead to the raising of various cytokine expressions in stromal cells [13-15]. And soon, it was confirmed to be the promoted factor in the carcinogenesis of several solid tumors, such as gastric cancer, pancreatic cancer, and lung cancer [16-18].However, its expression has not been reported in CRC metastasis. In our study, IL-32 expression in cancer cells was immunohistologically evaluated, and the clinical implications of IL-32 positivity with CRC metastasis were analyzed and discussed.

## Methods

### Patients and specimens

We retrospectively collected 70 consecutive patients with CRC, 47 cases of which were CRC organic metastasis lesions while the rest of which were primary CRC lesions (T4NxM0). All of them underwent surgery from 2000 to 2013 at Huashan Hospital, Fudan University, Shanghai, China. None of them received preoperative chemotherapy. The patient private identification information was deleted, and informed consent was obtained from all the patients. Clinical factors were assessed by the Medical Ethics Committee of Huashan Hospital.

### Immunohistochemistry

From paraffin blocks, the selected tissue was assessed on 4-μm sections on an automatic platform (Dako Immunostainer RM2145, Dako Corp., Copenhagen, USA). The samples were obtained from the 70 patients. Antigen retrieval was finished by microwaving in 10 mm citric acid monohydrate for 1 × 5 min at 900 W and for 3 × 5 min at 600 W. Sections were treated with 0.3% H2O2 for 30 min in order to block endogenous tissue peroxidase. The reaction was visualized by the Envision kit (Dako Corp., Copenhagen, Denmark) for IL-32 (ab37158, Abcam, Tokyo, Japan). The antibody was obtained from R&D Systems and used at the dilution of 1:200, being incubated with the tissue sections at 37°C for 1 h. Splenic tissue was used as a positive control for IL-32 expression. The positive control was included in each run. The optical density (OD) value of the tumor cell staining was measured for quantitative analysis.

Three experienced pathologists independently evaluated IL-32-stained slides without the knowledge of patient clinicopathologic information. The intensity and percentage of immunoreactivity was scored on a scale of 0 to 3+. Membrane staining in less than 10% of invasive tumor cells was scored as 0; faint/barely perceptive membrane staining was detected in 10% of tumor cells was scored as 1+; weak to moderate complete reactivity in more than 10% tumor cells or <30% was scored as 2+; strong complete reactivity in 10% or more of tumor cells was scored as 3+. A score of 0 or 1+ was considered negative while scores 2+ and 3+ were positive.

### Statistical analysis

Statistical analysis was performed by *χ*2 tests, and the results are considered to be statistically significantly different when *P* value is less than 0.05. The frequencies of variables are described in percentages. All analyses are performed using the SPSS version 19.0 software for Windows (SPSS Inc., Chicago, IL, USA).

## Results

### Samples

All 70 CRC patients were considered eligible and included in our analysis. Median age at time of metastasis resection or radical operation was 61 years (41 to 87); 47.14% patients were males and 52.86% females. The cases with other organic metastases include seven cases of peritoneal metastasis, four cases of ovarian metastases, three cases of great omentum metastasis, one case of gum metastasis, one case of osseous metastasis, and one case of bladder metastasis. Patients’ clinical characteristics are summarized in Table 1.

**Table 1:** **Clinical characteristics of the selected patients (** ***N*** **= 70)**

	***N***	**%**
Gender		
Male	33	47.14
Female	37	52.86
Position of lesions		
Primary lesions	23	32.86^a^
Brain	19	27.24^a^
Liver	11	15.71^a^
Other distant metastasis	17	24.29^a^

^a^The rate is calculated on the metastasis population.

### The relationship between IL-32 and organic metastasis of CRC

**Figure 1:**
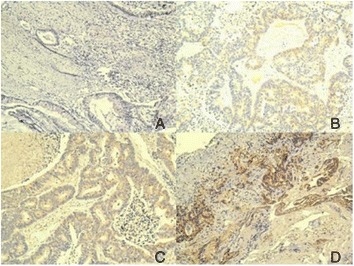
IL-32 staining scored 0 **(A)**; IL-32 staining scored 1+ **(B)**; IL-32 staining scored 2+ **(C)**; and IL-32 staining scored 3+ **(D)** magnification × 100.

Immunohistochemistry (IHC) IL-32 expression pattern is described in Table 2: 22 cases score 0 (31.44%), 12 cases score 1+ (17.14%), 18 cases score 2+ (25.71%), and 18 cases score 3+ (25.71%). Figure 1 shows the comparison of the IHC slides for IL-32.

**Table 2:** **IL-32 expression and immunohistochemical score of primary lesions and other metastasis**

**IL-32**	**Primary lesions**		**Brain metastasis**		**Liver metastasis**		**Other distant metastasis**	
	***N***	**%**	***N***	**%**	***N***	**%**	***N***	**%**
0	9	39.13	5	26.32	1	9.10	7	41.18
1+	7	30.43	2	10.52	3	27.27	0	0.00
2+	2	8.70	5	26.32	3	27.27	8	47.06
3+	5	21.74	7	36.84	4	36.36	2	11.76

Here, IHC IL-32 expression pattern of primary lesions is compared with brain metastasis, liver metastasis, and other metastasis. The results were considered to be statistically significantly different (*P* < 0.05), respectively, (Table 3).

**Table 3:** **Comparison of IL-32 expression between primary lesions and different metastases of CRC**

**Samples**	**Positive** ***N*** **(%)**	**Negative** ***N*** **(%)**	***P*** **value**
Primary lesions	7(30.44)	16(69.56)	0.0103
Brain metastasis	12(63.16)	7(36.84)	
Primary lesions	7(30.44)	16(69.56)	0.0013
Liver metastasis	7(63.63)	4(36.37)	
Primary lesions	7(30.44)	16(69.56)	0.0102
Other distant metastasis	10(58.82)	7(41.18)	

### The relationship between IL-32 and lymph node metastasis of CRC

Here, IHC IL-32 expression pattern of primary lesions with no lymph node metastasis was compared with primary lesions with lymph node metastasis. The results were considered to be statistically significantly different (*P* < 0.05), respectively, (Table 4).

**Table 4:** **Comparison of IL-32 expression between** ***N*** **−/** ***N*** **+ primary lesions of CRC**

**Samples**	**Positive** ***N*** **(%)**	**Negative** ***N*** **(%)**	***P*** **value**
No lymph node metastasis	1(7.69)	12(92.21)	0.0000
Lymph node metastasis	6(60)	4(40)	

**Figure 2:**
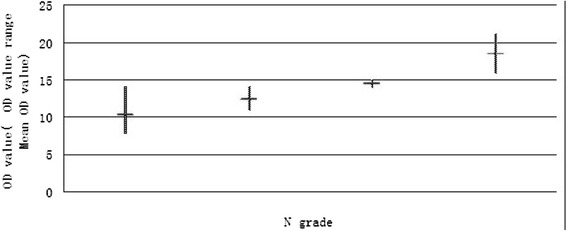
The relationship between *N* grade of primary CRC and OD value.

Here, the main OD values of different grades of lymph node metastasis (*N* grade, according to NCCN colorectal cancer practice guideline 2012) were compared altogether. The results showed that the OD value had the trend of rising with the level-up of *N* grade (Table 5, Figure 2).

**Table 5:** ***N*** **grade of primary CRC and mean OD value**

***N*** **grade**	***N***	**%**	**Mean OD value**
0	13	56.52	10.31
1	6	26.08	12.33
2	2	8.70	14.50
3	2	8.70	18.50

## Discussions

Several researches suggested that cytokines may be involved in the proliferation, metastasis, and preventing from antitumor cellular immunity [19-21]. Recent study showed that IL-32 can affect the signaling of NF-κB and STAT3 [22-24], which are confirmed to be the antiapoptotic and pro-angiogenic genes in cancer development [25]. Carmi *et al*. [26] suggested that IL-32 can promote angiogenesis and counterbalance the inflammation and antitumor immunity. Seo *et al*. [27] measured serum IL-32 levels of gastric cancer by ELISA and immunostaining, finding significantly higher levels of IL-32 to those of healthy volunteers. In this study, we demonstrate the association of between IL-32 and different metastases of CRC, aiming at finding new therapeutic direction for CRC metastasis.

### IL-32 and organic metastasis of CRC

IL-32 expression in tumor cells has been confirmed mainly occurring in the advanced stages of cancers [16,18]. But the relationship between IL-32 status and CRC organic metastasis has not been clarified. Our research aimed to describe the influence of IL-32 on CRC organic metastasis, to compare the IL-32 expression in primary lesions or organic metastasis, so as to find the biological mark of CRC organic metastasis.

As determined by our analysis of 70 CRC patients, IL-32 expression correlated with the organic metastasis of CRC. The rate of IL-32-positive cases of CRC primary lesions was 30.4%, while brain metastasis 63.16%, liver metastasis 63.63%, and other organic metastasis 58.82%. The positive rates of CRC brain metastasis, liver metastasis, and other distant metastasis were similar. There were great differences between the results of the rate of IL-32-positive cases of CRC primary lesions and different organic metastases (Table 3). As a result, IL-32 supposed to be the nonspecific mark of CRC organic metastasis.

### IL-32 and lymph node metastasis of CRC

It has been suggested that IL-32-positive expression was frequently associated with lymph node metastasis in gastric cancer and lung cancer [16,18]. But there is still no evidence whether there is association between IL-32 expression and CRC lymph node metastasis. Our research is here to analyze the IL-32 expression staining in CRC samples (T4NxM0), so as to check whether IL-32 relates to CRC lymph node metastasis.

The result showed that the rate of IL-32-positive cases of CRC lymph node metastases was 60%, much higher than those without lymph node metastasis. The results were considered to be statistically significantly different (Table 4). The tendency showed that the level of IL-32 expression may influence the *N* grade of CRC (Table 5, Figure 2). Further study with larger sample size of the primary lesion population should be made for exactly evidence.

## Conclusions

In conclusion, this research raises the concept of an important role of IL-32 in CRC. Over-expression of IL-32 may stimulate the organic metastasis and the lymph node metastasis of CRC. IL-32 was confirmed to be the biological mark for CRC metastasis. In CRC, this result may promote the therapeutic potential for CRC metastasis. However, there are still several aspects of IL-32 remaining elusive. Our study also presents some limitations. The follow-up of the patients has not finished yet. Further analysis on larger populations is required to support the results of this study. And also, further researches should be taken out to clarify the underlying pathway of IL-32, so as to propose a new clinical therapy for CRC metastasis.
